# Rice Crop Counting Using Aerial Imagery and GIS for the Assessment of Soil Health to Increase Crop Yield

**DOI:** 10.3390/s22218567

**Published:** 2022-11-07

**Authors:** Syeda Iqra Hassan, Muhammad Mansoor Alam, Muhammad Yousuf Irfan Zia, Muhammad Rashid, Usman Illahi, Mazliham Mohd Su’ud

**Affiliations:** 1 Department of Electronics and Electrical Engineering, Universiti Kuala Lumpur British Malaysian Institute (UniKL BMI), Batu 8, Jalan Sungai Pusu, Gombak 53100, Malaysia; 2 National Centre for Big Data and Cloud Computing, Ziauddin University, Karachi 74600, Pakistan; 3 Department of Electrical Engineering, Ziauddin University, Karachi 74600, Pakistan; 4 Faculty of Computing, Riphah International University, Islamabad 46000, Pakistan; 5 Faculty of Computing and Informatics, Multimedia University, Cyberjaya 63100, Malaysia; 6 Malaysian Institute of Information Technology, University of Kuala Lumpur, Kuala Lumpur 50250, Malaysia; 7 Faculty of Engineering and Information Technology, School of Computer Science, University of Technology, Sydney 2006, Australia; 8 Department of Computer Engineering, Umm Al Qura University, Makkah 21955, Saudi Arabia; 9 Department of Electrical Engineering, FET, Gomal University, Dera Ismail Khan 29050, Pakistan; 10 Water and Engineering Section, MFI, Universiti Kuala Lumpur Malaysian France Institute (UniKL MFI), Section 14, Jalan Damai, Seksyen 14, Bandar Baru Bangi 43650, Malaysia

**Keywords:** aerial imaginary, GIS, rice crop, soil testing, UAV, yield estimation

## Abstract

Rice is one of the vital foods consumed in most countries throughout the world. To estimate the yield, crop counting is used to indicate improper growth, identification of loam land, and control of weeds. It is becoming necessary to grow crops healthy, precisely, and proficiently as the demand increases for food supplies. Traditional counting methods have numerous disadvantages, such as long delay times and high sensitivity, and they are easily disturbed by noise. In this research, the detection and counting of rice plants using an unmanned aerial vehicle (UAV) and aerial images with a geographic information system (GIS) are used. The technique is implemented in the area of forty acres of rice crop in Tando Adam, Sindh, Pakistan. To validate the performance of the proposed system, the obtained results are compared with the standard plant count techniques as well as approved by the agronomist after testing soil and monitoring the rice crop count in each acre of land of rice crops. From the results, it is found that the proposed system is precise and detects rice crops accurately, differentiates from other objects, and estimates the soil health based on plant counting data; however, in the case of clusters, the counting is performed in semi-automated mode.

## 1. Introduction

Precision farming is observing, measuring, and responding to a system that sustains the structure used for managing the farm and producing the extreme yield in existing resources [1]. Outdated approaches such as combined pest management used in farming are inadequate, and the utilization of synthetic pesticides affects animals, human beings, and also the environment [2]. As a society of the third millennium, humankind has started to master every aspect of its day-to-day life, making it more efficient in terms of time and resources. One of the most critical aspects of human life is nutrition, and we can notice severe advancements in the agricultural sector that satisfies these human needs. Especially within developing countries, over seventieth of agricultural individuals depend upon the agriculture fields [3].

Improvements such as precision farming, fertilizers, etc., are helping to utilize the earth’s resources most efficiently, but there is still much room for advancement. Consequently, in the present era, there are several developments in agriculture for growing crop productivity.

Among several other important crops, over half of the world’s population uses rice as their main source of energy because it is a complex carb. No other crop provides as much food, as much employment for farmers, or as much support for the environment. For 70% of the impoverished in Asia, for whom rice is frequently the only source of income, it is much more than just a food crop [4]. The amount of protein, iron, manganese, fiber, and vitamin B in rice varies depending on the strain. It can thus be quite important in the fight against malnutrition. Therefore, monitoring and counting rice crop helps us to increase yield, identify soil health, and save the crop from diseases.

While the number of rice seedlings within the field is one of the most agronomical elements for defining rice yield, this investigating task, however, remains chiefly performed manually. It includes the exploitation of human vision instead of computer vision and is so cumbersome and long [5]. Therefore, a quick and correct technique is needed for the observation of the potency of crop supervision practices. Furthermore, it should include the pre-estimations for the yield of rice crops and can be employed as a phenotyping feature in breeding plans.

Developed countries have already started the use of unmanned aerial vehicles (UAVs) in their precision agriculture. Automation pilotless aeronautical vehicles (UAVs) [3,6], normally referred to as automatons, work as driverless airplane frameworks. The UAVs are utilized in various applications such as mechanical checking, photography, reconnaissance, air emergency vehicles, conveyance, and many more [7,8,9]. In other words, the agricultural sector has a well-defined task for this new progress in the computer vision sphere—Crop counting for better yield prediction, segmentation of problematic crop areas such as plants beaten down due to weather activity, flooded regions of the fields, detection of plant diseases, etc. Solving described tasks allows farmers to better prepare for upcoming dangers to their yield, minimize their losses and maximize harvest and profits.

Farmers face many problems during the crop cycle as they do not have sufficient water, lesser use of modern equipment, dependencies on traditional farming techniques, poor storage facilities, transportation problems, high-interest rates, and government schemes [10]. The solutions to the problems are, adopting modern farming, educating farmers, crop insurance, and better water management. One of the probable solutions to these challenges can be crop counting to increase crop yield and identify soil health. Identification of soil health helps to indicate fertile and barren land, which affects crop productivity. Furthermore, the crop counting solution should be easily adaptable, cost-effective, and reliable. In other words, there is a need for a system that can identify the crop count and may indicate the soil fertility by comparing its expected yield according to the land capacity.

In this research, there is a tendency to propose an associated economical technique that uses computer vision to accurately count rice seedlings during a digital image. First, an associate UAV equipped with red–green–blue (RGB) cameras acquires field pictures at the phanerogam stage. The UAV flies over the rice fields for approximately 10 min and captures imagery data. It takes a total of 8 flights. Next, it employs an ArcGIS to regress the density map and estimate the number of rice seedlings for a given UAV image. ArcGIS is a geographical information system (GIS) application that enables the handling and analysis of geographic data by visualizing geographic statistics through layer-building maps, such as climate data or trade movements. Subsequently, rice crop counting is performed to identify soil health and indicate fertile or barren land. The area planted with rice is classified by employing the Iso cluster unsupervised classification technique using the ArcGIS program. 

This research contributes a novel data set with a customized technique of using a combination of UAV and ArcGIS. It is based on a deep learning model to estimate crop yield. Furthermore, it indicates soil health based on productivity. In this context, we have monitored two different regions of rice crops. In the first region, the monitoring of rice crops is performed after 1 week of sprouting. The region is 4 acres (1 acre of mechanical planting and 3 acres of manual planting). In the second region, the monitoring of 35 acres (mechanical planting) is performed. The monitoring of the field is performed using a UAV. The aerial imagery, captured by UAV, is processed with ArcGIS for plant counting. The ArcGIS is used for pointing the coordinates to map the data on the image.

The proposed solution helps to identify the crop count and indicate soil health. This research also helps to indicate fertile and barren land. Consequently, it helps to take all the necessary steps to make barren land fertile for the next agriculture cycle. The result aids farmers or landmen in taking safety precautions for increasing yield and treating land according to its health. The research is based on identifying crop yield and identifying soil health. The output of the proposed system is 0.99 percent efficient (in terms of accuracy).

This article comprises six sections. Section 1 provides the introductory information and its importance in today’s world. Section 2 is the literature review that provides the background of the study work and related work. Section 3 illustrates the material and methods used in the research. The experimental evaluation is presented in Section 4. Section 5 presents the result and discussion, and finally, the article is concluded in Section 6.

## 2. Literature Review

This section comprises two sections. In Section 2.1, the background of the research is presented, and Section 2.2 provides details of the work related to the research.

### 2.1. Background

Each touchpoint in our life today is being etched with a new deoxyribonucleic acid (DNA), thanks to the data and technology. The barriers between the physical, digital, and biological domains are disappearing as we live and breathe, thanks to the fourth industrial revolution and its convergence of technology.

The fourth industrial revolution [11] is preparing the way for huge changes across all industries, but more especially in healthcare and life sciences, with its storm of big data and digital technologies. The four key pillars that life sciences organizations must adhere to in order to succeed in the fourth industrial revolution in terms of patient centricity, commercial efficiency, and overcoming regulatory obstacles.

- Industrialization Analytics
- Data Integration
- Collaborative Cross-functional
- Augmented Intelligence

By increasing the efficiency of farming operations by increasing agricultural production, reducing environmental impact, and automating farmers’ work, they contribute to the growing body of knowledge about the potential role of blockchain technology in promoting the idea of smart farming [12] UAVs [13] natural environments and Artificial Intelligence (AI). In [14], look for water resources in places where a satellite would not typically be able to acquire photographs, and the utility of such an autonomous flying IoT is proven.

The experimental work satisfies the demands of automatic and real-time environmental parameter monitoring by employing both above- and below-ground sensors [15]. In this article, the fourth industrial revolution technologies, such as smart farming, natural environments, and UAV, along with their AI applications, emerged to produce a sustainable solution for soil health and crop count.

#### 2.1.1. Smart Farming

The term “smart farming” refers to the management of farms using the Internet of Things (IoT), robotics, drones, and AI to improve product quantity and quality while minimizing the amount of human labor needed for production. A growing number of people are becoming interested in smart farming technologies and precision agriculture [16] because of their potential to satisfy this rising demand and meet the needs of the world’s food supply. To improve crop productivity and food product quality, smart farming technologies integrate technology and data-driven agriculture applications. Globally, there are many examples of smart farming use cases [17] to [18] that show the effects of this new way of doing agriculture.

#### 2.1.2. Unmanned Aerial Vehicle (UAV)

Another ground-breaking innovation with enormous promise in precision agriculture is the use of unmanned aircraft systems (UAS) as sensing and/or communication platforms [19]. It was developed as a low-cost alternative technology for acquiring images, monitoring the environment, and achieving high spatial and temporal resolution. UAV use in agriculture is growing at the moment to help farmers with monitoring and decision making on the farm [20]. UAS are used in a variety of agricultural applications, including weed control, pesticide application, fertilizing, and irrigation. Additionally, the integration of UAS technology with cutting-edge 3D reconstruction modeling methods has made it possible to monitor the crop’s growth metrics at the plant level. A new and exciting era of agriculture-food production is being ushered in by the integration of several major emerging technologies into the agricultural sector.

#### 2.1.3. Artificial Intelligence (AI)

In agriculture, there is a quick adaptation to AI in its various farming techniques. The concept of cognitive computing is one that imitates human thought processes as a model in the computer. This results in turbulent technology in AI-powered agriculture, rendering its service in interpreting, acquiring, and reacting to different situations (based on the learning acquired) to enhance efficiency. To harvest benefits in the field by catching up with the recent advancements in the farming sector [21].

Recent improvements in the accessibility of pertinent data, processing, and algorithms have enabled AI to start delivering on its promise of creating real value. In this essay, by enhancing what is being detected and monitored, the most immediate application will be to increase the accuracy of information about what is happenning on the farm. This has the effect of providing farmers with more precise warnings [22].

### 2.2. Related Work

One of the typical employments for UAVs in precision farming is found in [23], where aerial images were collected during the survey over four days in the test field. Consequently, the convolution neural network (CNN) was designed and trained to identify the three-dimensional location of cotton. Finally, the cotton was detected from random pictures. In comparison with manually, the model counts faults 3–4 flowers for the field with a solitary plant in each plot. The proposed technique in [24] is actualized on a 10-week-old spinach plant utilizing computer vision—Excess Green Index and Otsu’s strategy—and move to get the hang of utilizing convolutional neural systems to distinguish and tally plants, but it is not able to distinguish accurately due to object confusion.

The calculations dependent on deep neural systems are proposed to recognize tobacco plants [25], corn plants [26,27], and banana plants [28] in pictures caught by automated aerial vehicles (UAVs). These UAV pictures are portrayed by a high spatial goal. The profound learning-based methodology can precisely include plant seedlings in the field. Seedling recognition models prepared in this investigation and the commented-on pictures can be utilized by the exploration network and the cotton business to advance the improvement of answers for the seedling outcome and count [29].

The work in [30] presents an approach dependent on otherworldly lists and advanced picture examination to perform populace, including in sunflower plants. Results demonstrate that it is conceivable to gauge the number of plants in the picture with an error of 10%. The relationship between the ground cowl and carefully checked plants was horrendously low. The work of UAVs and picture processes can improve ranch executives and help field experimentation for science and rearing functions [31]. Their investigation was to create and approve vigorous field-developed apple nursery plant tallying calculation that depends just on height pixel estimations of little UAS-based low elevation RGB symbolism information. The nursery field pictures were acquired from exploitation; little UAS worked at thirty on the head of the base level. Picture preparation, which is acquired by UAS, was acted in a GIS programming [32] and [33] presents a data frame structure that is made for automatic plant count and for increasing plant phenotyping datasets that utilize delivered pictures of engineered plants is proposed in [34]. They show that genuine and engineered plants are fundamentally exchangeable when preparing a neural system for the leaf count task [34].

The technique used in this article indicates the health of land according to the density of growing seedlings, and the gaps show the ungrown area of land. The research in the same area is presented below in Table 1.

In [35], the authors have used the UAV and YOLOv4 model for the quick detection of rice ear, which can detect rice fields in different states of health with the achievement of Map 95%. In their research, rice ears overlapping leads to missed identification. In [36], authors used CNN and multispectral images from UAV for quantity estimation of citrus trees, which is capable of counting and geolocation, but it compromises efficiency in the land of the highly populated region. In [37] combination of UAV and YOLOv2 models was used for the quick detection of green mangoes, and the average mean precision of the model is 86.4%, with an error rate of 1.1%.

In [34], L-system modeling (Deep learning) is used for the identification of rosette plants, and their system identifies the rosette plants with the accuracy of %, but the model is confused with other objects due to less data size. In [38], Olive trees are monitored and identified using U2 net-deep learning network. The model is accurate with an error rate of approx. 15% due to the complex model. The technique proposed in this article is based on a deep learning model which utilizes the imageries from UAV and is further processed on ArcGIS software which is a very user-friendly, cheap, and easy way to estimate the crop yield and indicate the soil health based on production rate. It requires good-quality images for better detection of crops.

## 3. Materials and Methods

This section is divided into two sections. In Section 3.1, the details of the experimental site and imaging device specifications are presented. Section 3.2 explains the rice seedling counting dataset.

### 3.1. Experimental Site and Imaging Devices

The study was carried out in Tando Muhammad Khan, Sindh, Pakistan, in June 2020. A total area of 40 acres was chosen for the study and divided into two regions. Region 1 (5 acres) (25°08′16.4″ N, 68°35′26.5″ E) and region 2 (35 acres) (25°08′12.9″ N, 68°36′08.9″ E). For the acquisition of images, a DJI Phantom 4 Pro drone was used. The UAV, flight assignment, and specifications of the camera are shown in Table 1 and Table 2. A predefined flight plan was developed using a mission planner Map Pilot for DJI software. The UAV flew autonomously over the rice field at 35 m. To eliminate the chance of data distortion and to stabilize the flight during image acquisition, the experiment was performed in June 2020 on a sunny day in considerably low winds. The images were acquired with the overlap of 70% Along Track and 60% Across Track to fulfill the total coverage of the study area.

**Figure 1:**
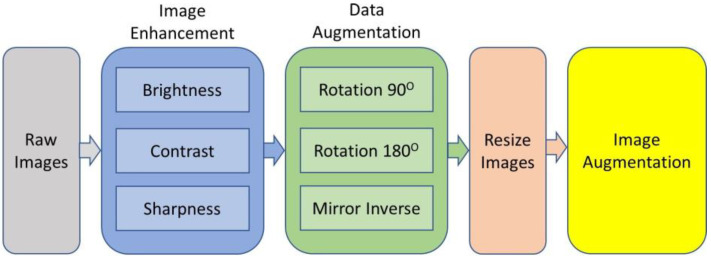
Preparation of dataset.

Figure 1 explains the flow of data collection. After hovering, the composed images were processed in the Agisoft Metashape Professional software ver. 1.5.4 (2019), which, through its algorithms, allowed to orient of the images and generated the ortho mosaic, digital terrain, and surface models (DSM). These models were later analyzed in a GIS environment. 

The UAV used in the experiments has a 1388 g weight with a LiPo battery capacity of 5870 mAh. It can fly for approximately 10 min at an altitude of 35 m. The total eight flights of the UAV are used for data collection for the duration of 80 min, as shown in Table 2 below.

The camera specification for monitoring fields for estimating yield and indication of soil health is represented in Table 2. The Sensor has a 1″ CMOS effective pixel with mechanical and electronic shutter speeds of 8–1/2000 s and 8–1/8000 s, respectively, and supports JPEG and DNG(raw) photo formats, as mentioned in Table 3.

### 3.2. Rice Seedling Data Set

The imagery data set of rice seedlings is about 1200 RGB images using an unmanned aerial vehicle. The imagery data set consists of images of rice seedlings that are manually and mechanically planted over 40 acres of land. Table 4 shows the details of the imagery data set.

**Figure 2:**
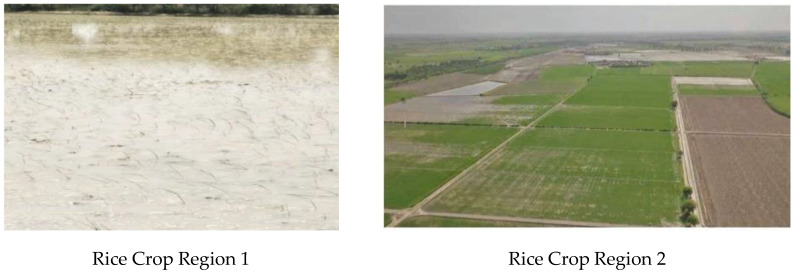
Seedling plantation in region 1 and region 2.

Region 1 (35 acres) (25°08′16.4″ N, 68°35′26.5″ E) and region 2 (approx. 5 acres) (25°08′12.9″ N, 68°36′08.9″ E) are shown in Figure 2.

**Figure 3:**
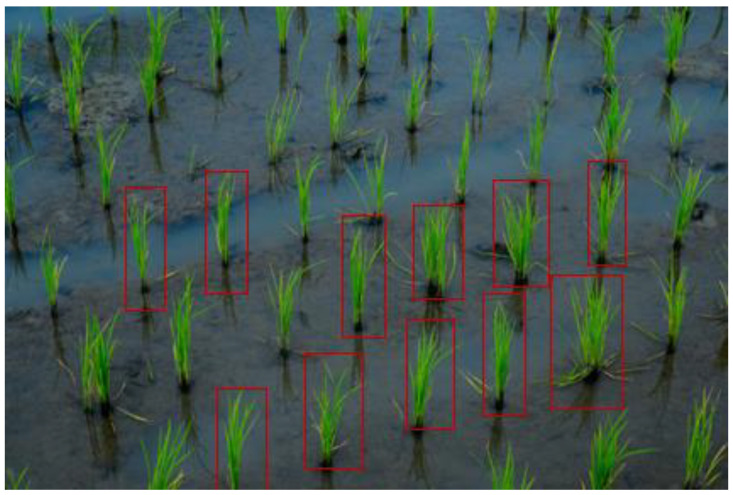
Training samples for extracting features.

Figure 3 illustrates the training samples for extracting features. The red bounding boxes show the rice training sample based on the system detecting rice crops. Once the image is captured, the next step is to prepare a test and translate the image into the configuration of a deep learning model that has to be given descriptions of images of rice plants or seedlings so that it can identify comparable pixels in order to understand what it is supposed to be detecting. Creating realistic training assessments is essential when teaching a profound learning model or any picture grouping approach. Additionally, it is typically the longest advancement overall. To give a deep learning model the information it requires, rice plants or seedlings must be highlighted to show the algorithm their potential sizes, shapes, and marks, as shown in Figure 3.

These highlights enhanced a certain organization called picture chips’ deep learning methodology. Picture chips are tiny symbolism-filled squares that were sliced from the original image. It will be easy to trade them as image chips with metadata once they have achieved a sufficient number of highlights in the image classification board. The detection objects using a deep learning device in ArcGIS Pro 2.5, which relies on well-established learning structures, can be prepared using these preparatory tests.

The aim of this research is to provide a sustainable and easy method of identification of crop yield and soil health in terms of productive or barren lands. The use of aerial vehicles provided ease in monitoring and capturing the data for the identification of soil health and crop yield by counting them and estimating the results by mapping according to acres. This research is carried out in the area where mechanical plantation and manual plantation are both carried out, and it has to find out the number of crops and estimation of soil health.

**Figure 4:**
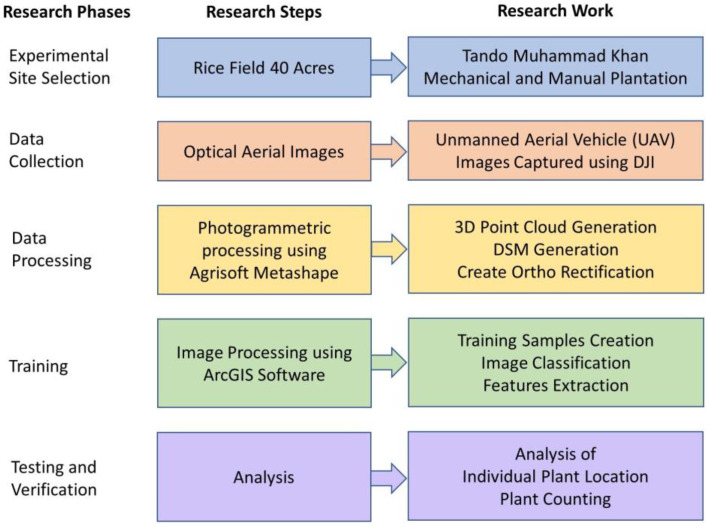
Block diagram of the research work.

In Figure 4, the research is presented as data collection is carried out in rice fields. The area of the rice field is about 40 acres, where the mechanical and manual plantations both are carried out. The experimental site is at Tando Muhammad Khan, where the UAV flew to collect imagery data of the field. The data are the optical images that are processed to remove outliers and garbage data for image processing. Training Samples Are created, and image classification is performed to train the model for selecting individual plant locations and counting plants as well.

**Figure 5:**
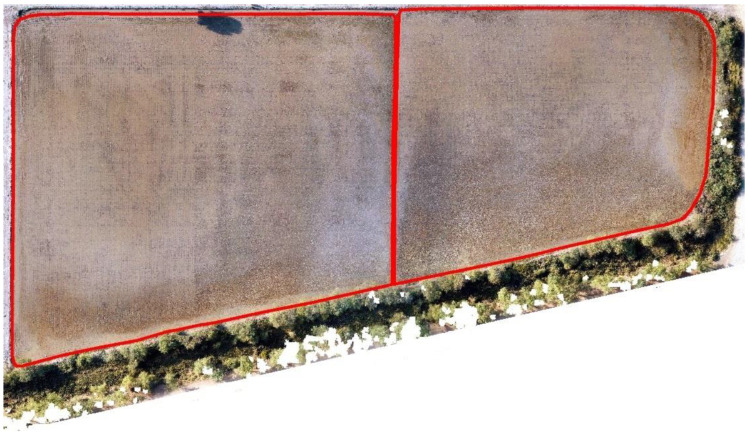
Mechanical and manual plantation in region 1.

Inventorying each rice plant on the field manor would take a great deal of time and an enormous workforce. To improve the procedure, you′ll utilize a profound (deep) learning model in aeronautical reconnaissance coverage geographic information system (ArcGIS) programming to distinguish rice plants. The principal step is to discover symbolism that has a fine enough spatial and unearthly goal to distinguish plants and seedlings. The plantation is carried out mechanically and manually. Firstly, in region 1, point out mechanical plantation and manual plantation areas, as shown in Figure 5.

**Figure 6:**
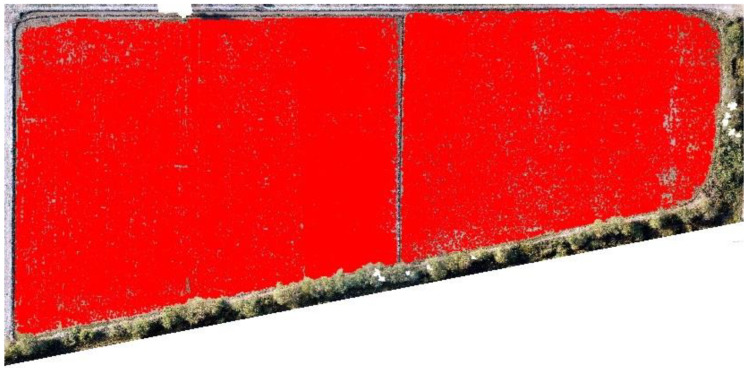
Point demarcation of region 1.

After discrimination of mechanical and manual plantation areas, the next step is to perform point demarcation of plants or crops, which indicates the growing rice crop as shown in the figure below. Figure 6 shows the point demarcation of the mechanical and manual of region 1.

**Figure 7:**
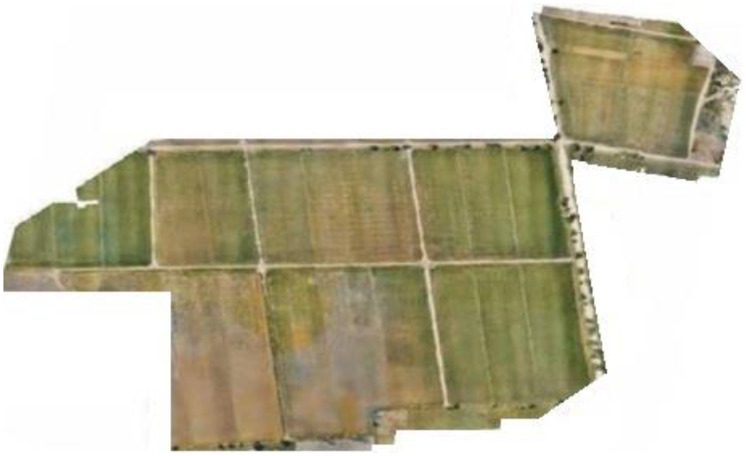
Image shows the mechanical plantation of region 2.

Ortho-amended pictures were produced utilizing the boundaries that appeared in Table 3 with a tagged image file format (TIF) augmentation: 10 mm/pixel picture for the 35 m flying stature. Plant point demarcation helps us to identify live plants and dead plants, which help to estimate the rice yield and indicates the land or soil health as to whether it is productive or barren. To clarify further, below are the separate images of mechanical and manual images of region 1. Figure 6 shows the mechanical plantation, and Figure 7 shows the point demarcation of region 4.

**Figure 8:**
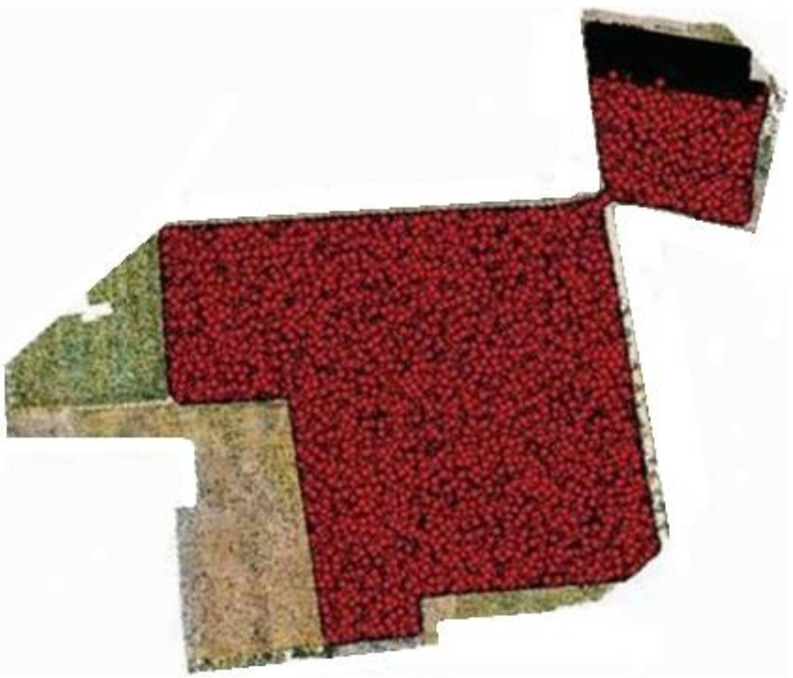
Point demarcation of region 2.

Region 2, which is approx. 5 acres and is geolocated at 25°08′12.9″ N, 68°36′08.9″ E, carried only mechanical plantation, and below is the point demarcation of region 2 to point out live plants for estimating yield according to acers and indicate soil health concerning dead plants either the soil is productive or barren. Figure 8 shows the point demarcation of region 2.

Once having the imagery, at that point, prepare tests and convert them to a configuration that can be utilized by a profound learning model. For the model to perceive what it’s entrusted with finding, it has to characterize pictures of rice plants/seedlings so it can recognize comparative pixels. Making reasonable training tests is vital once instructing a profound learning model or any picture grouping model. It is additionally frequently the most tedious advance all the while. To furnish a profound learning model with the data, it needs to extricate all the rice plants or seedlings in the picture and make the highlights for various rice plants or seedlings to show the model the size, shape, and mark of rice plants or seedlings. These preparation tests are made and overseen through the Label Objects for Deep Learning instrument.

While capturing samples of plants in the territory, highlights were digitized all through the picture. These highlights added something extra to the profound learning model in a particular organization called picture chips. Picture chips are little squares of symbolism cut from the source picture. When they have made an adequate number of highlights in the Image Classification board, it will be anything but difficult to trade them as picture chips with metadata. These preparation tests can be utilized to prepare a model utilizing the Detect Objects utilizing a Deep Learning device in ArcGIS Pro 2.5, which depends on profound learning structures, for example, TensorFlow, Keras, or CNTK. To introduce these profound learning libraries, clone the default Python condition utilizing the Python Command Prompt.

**Figure 9:**
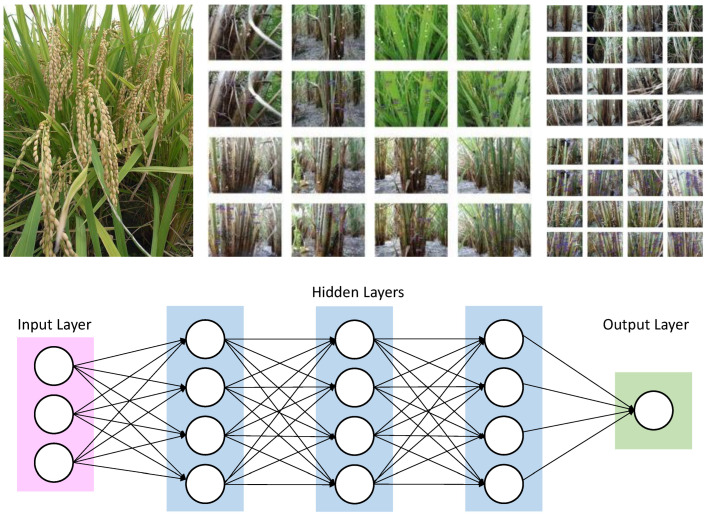
Deep learning architecture.

The Train Deep Learning Model geoprocessing apparatus utilizes the picture chips that are marked to figure out what blends of pixels in a given picture speak to rice plants. Figure 9 shows the deep learning architecture. The preparation procedure delivers an Esri model definition (.emd) record that can be utilized by other profound learning instruments inside ArcGIS. At that point, the populated of (.emd) record and utilize the Detect Objects Using Deep Learning instrument to recognize rice plants or seedlings in the picture.

Geospatial software called ArcGIS is used to view, edit, manage, and analyze geographic data. For mapping on PC, mobile, and the web, Esri creates ArcGIS. The science of where is their slogan. As a result, location intelligence and analytics are the main focus of ArcGIS. Professional software called Agisoft Metashape analyses digital photos, generates digital models and point clouds for 3D spatial data, and combines snapshots to produce orthogonal images. Additionally, the software’s features include the ability to create visual effects and take indirect measurements of things at different scales.

Agisoft Metashape Professional Software is based on a deep learning architecture. The background of their inputs and producing output depends on deep learning. The input image is integrated with a set of K kernels.

(1) $$Mk\in D_{V\times V},\;\;k=1,\;2,\;\ldots ,\;K$$

then biased

(2) ak∈D,  k=1, 2, …,K

are applied

Each of these operations creates a new feature map ak through an element-wise non-linear transform σ(·). For hidden layers, the same procedure is conducted.

(3) Plk=σ(Mlk ⊗ Pl−1+alk)

where the symbol ⊗ stands for the discrete convolution operator, and its particular type of operation can take several other forms, including “valid”, “same”, “extra”, “strided”, “fractional-strided”, and others.

**Figure 10:**
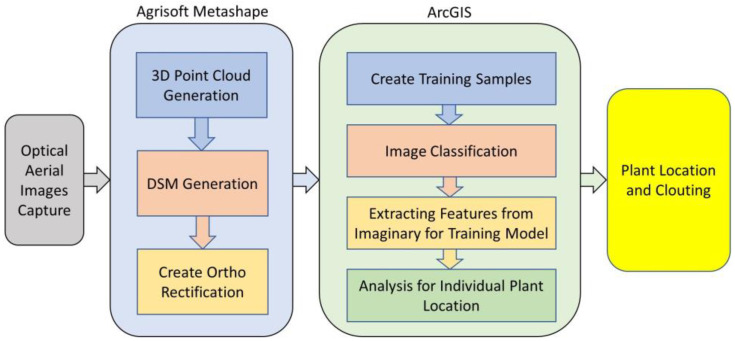
Workflow Chart.

The system block diagram explains the working model adopted in this work. In Figure 10, the workflow chart of this research is presented as images captured using UAV, and then the images are processed with AgiSoft Metashape Professional Software (AMPS). In AMPS, ortho rectification is generated by 3D point and DSM generation. After ortho rectification, training samples are created through which image classification is performed, which extracts features for the training model. Plant location and plant count are identified by the analysis of location. This flow of work helps to identify the location-based plant count from which the highly populated, less populated, and barren land can be identified easily.

## 4. Experimental Evaluation

**Figure 11:**
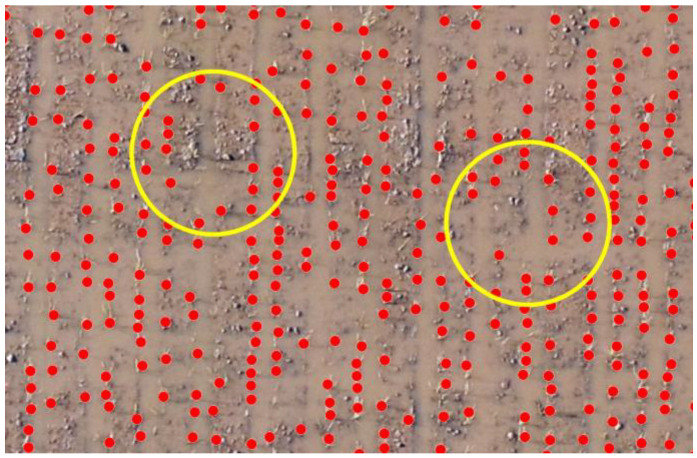
Identification of gaps in the mechanical plantation at scale 1:30 in region 1.

The experiment is carried out in Tando Muhammad Khan in an area of 40 acres where both mechanical and manual plantation is carried out. Drone DJI is used for monitoring and capturing images from fields of rice. A predefined flight plan was developed using a mission planner Map Pilot for DJI software. The UAV flew autonomously over the rice field at 35 m and captured images, and monitored fields to identify the land condition and health. The experimental findings and the outcomes of the proposed technologies are explained in this section. The plant count of mechanical and manual plantation is presented below in region 1. Figure 11 shows the identification of gaps in the mechanical plantation of region 1. The gaps shown in Figure 10 show that there is no plantation of rice in the gap area.

**Figure 12:**
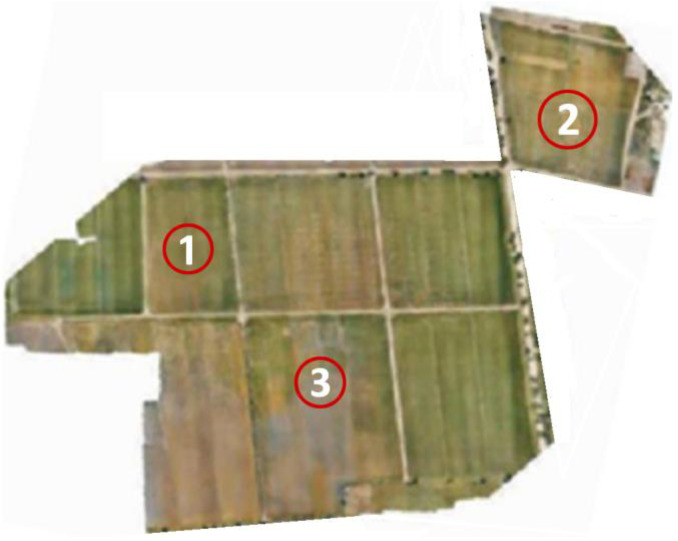
Areas of interest are labeled as (1, 2, and 3) in region 2.

Figure 12 shows the selected areas labeled as 1, 2, and 3 for counting plants. These are the areas where monitoring of rice fields is carried out, and it is considered region 2. The mechanical plantation is carried out in region 2, and the rice seedling is well organized in this region which helps to identify the gap more accurately.

**Figure 13:**
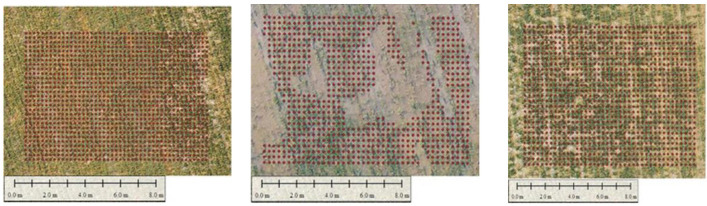
Sowing plantation pattern at areas (1,2, and 3) in region 2.

Figure 13 shows the sowing pattern of plants in areas (1, 2, and 3) of region 2. These plants are mechanically grown, and a dotted pattern shows the growing areas, and empty spaces indicate the problematic area. The images in Figure 11 show the point demarcation of red color dots on the field, which shows the fertile and barren region of land.

**Figure 14:**
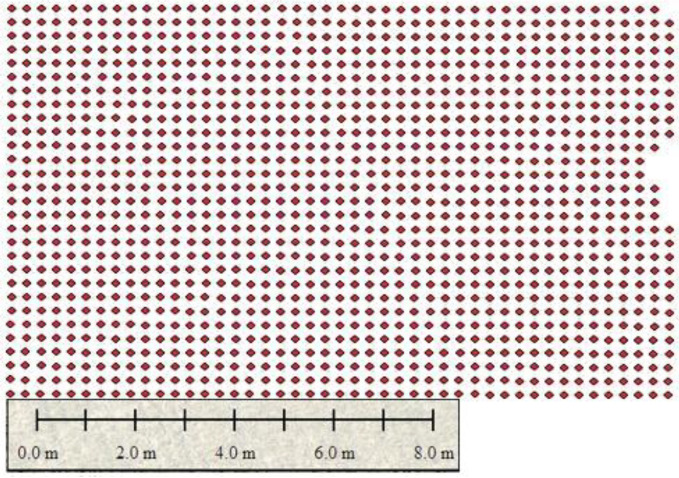
Plantation is uniform in area 1.

**Figure 15:**
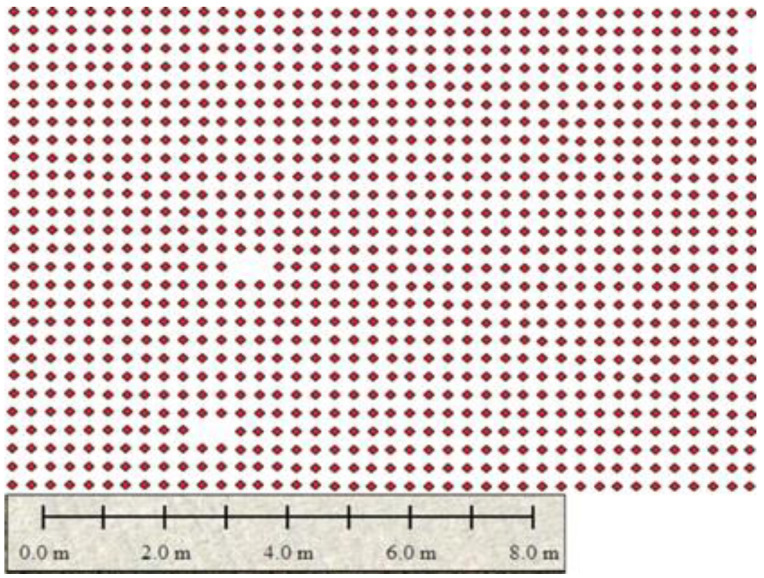
Plantation is uniform in area 2.

**Figure 16:**
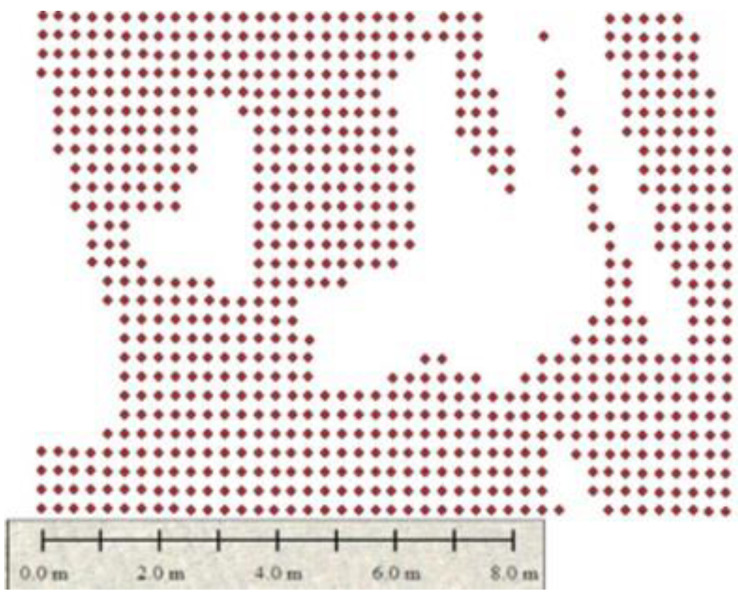
Plantation has extreme gapping in area 3.

Figure 14, Figure 15 and Figure 16 show the identification of gaps in the plantation of areas (1, 2, and 3). At this stage, the pattern of plantation can be identified by estimating crop yields by counting them and also indicating the problematic areas where plants are not growing. Figure 12 shows that the plantation is uniform in area 1 of region 2.

The mediocre gapping in the plantation in area 2 is visible in Figure 15. The gaps are identified precisely, and counts are verified according to the per acre land grown density.

Finally, Figure 16 shows the extreme gapping at area 3 of region 2, which is the most problematic region where many gaps can be visualized easily. This highlights the infertility of the land, which is needed to be treated before farming in the next session, which can save assets and time.

## 5. Results and Discussion

The rice crop originates in tropical lowlands and needs an extended heat season. Moreover, it is grown up where the night-time temperatures keep on the top of sixty degrees for a minimum of 3 months of the year. Furthermore, it is grown in water. Therefore, there is always water on land where the rice is growing. Consequently, to avoid reflection, early photography before the sun and after 1:30 pm is carried out for the data collection. If the data are required to collect at any time, the reflector camera (FLIR Vue Pro, Arduino Camera OV7670, 5MP Raspberry pi camera module, etc.) is placed with the drone camera, which avoids the reflections in the image. A drone camera is used for collecting images of rice fields which is processed for learning and labeling rice crops, and then ArcGIS is used for counting them

The maximum yield per acre rice field is from 80,000 to 150,000 plants. The amount of rice calculated is shown in Table 4 and Table 5, which is below the per-acre yield because the soil is loam. This research is to identify soil health by crop counting. The Arc GIS is used to estimate the crop yield, which leads to identifying soil health. The model is 0.99% accurate and capable of distinguishing crops from other objects, as it is also verified by an agronomist. The total plant counts of mechanical and manual plantation are presented in Table 5.

To obtain maximum paddy yield, from 80,000 to 150,000 plants are recommended per acre. This can be accomplished by preserving a plant-to-plant and row-to-row separation of 22.5 cm or 9 inches. However, in farmer’s fields plant populace of 60,000–65,000 has been detected [39]. Table 6 (plant counting mechanically) presents the total and per-acre count.

The aim of this research is to provide a sustainable and easy method of identification of crop yield and soil health in terms of productive or barren lands. The use of aerial vehicles provided ease in monitoring and capturing the data for the identification of soil health and crop yield by counting them and estimating the results by mapping according to acres. Table 7 provides a detailed comparative analysis of results obtained utilizing the existing techniques.

In [35], with the growing availability of RGB data with extremely high spatial resolution, this study demonstrated the efficiency of a deep convolutional neural network technique for producing rice density prescription maps using UAV-based imagery. For counting and geolocation, their solution in [36] performs noticeably better than competing object detection techniques. The outcome showed in [37] how well the algorithm detected green mangoes and offered a methodological guide for an instantaneous calculation of the number of green mango fruits in plantations. The modeling of complete crop plots is one possible area of significance [34]. The results of this study [38] show that the method of UVA RGB pictures and the U2-Net model may offer a very accurate and robust extraction result for olive tree tops and is useful in the dynamic monitoring and management of orchard trees. Techniques. Table 8 provides the outcome of the proposed technique.

The technique used in this article is a combination of UAV, AgiSoft Metashape Professional, and ArcGIS with deep learning. The UAV is used to gather imagery data, which are then processed to extract features on AgiSoft Metashape Professional software. Subsequently, ArcGIS is capable of counting the rice crop based on deep learning. Table 8 shows that the proposed technique is implemented on rice crops over an area of 40 acres where both plantations (manually and mechanically) exist. The total experimental count is 2,445,546, which is below the expected outcome of 3,200,000–6,000,000 because the land is loam or baren. The result is further verified by agronomists who declared that the soil is infertile.

This outcome helps the farmer to treat the affected area of land, which in turn saves assets. The assets include the sprays of medicines required to treat the land or water. These sprays only apply to the area of land where the crop is not grown and minimum growing areas. Therefore, the proposed system is cost-effective, saves assets, and reduces human efforts. To summarize, the agricultural sector has a well-defined task for this new progress in the computer vision sphere—crop counting for better yield prediction, segmentation of problematic crop areas such as plants beaten down due to weather activity, flooded regions of the fields, detection of plant diseases, etc. Solving described tasks allows farmers to better prepare for upcoming dangers to their yield, minimize their losses and maximize harvest and profits.

## 6. Conclusions

In this research, the counting of rice crops using aerial imagery was performed to increase the yield. Aerial images of the rice crop were captured in the area of 40 acres of rice crop in Tando Adam, Sindh, Pakistan, using a UAV equipped with a GIS system. The results show that the per acre count is less than the standard count of rice per acre in some areas due to the soil, not loam which affects the seeds. The findings are that mechanical plantation is an effective way of growing plants and increasing yield count. The results are due to improper soil that affects the overall count of regions. The software identifies the plant based on specified width and height, which is later checked via manual quality control. In the quality control process, visual interpretation is conducted based on the pattern, the cluster size, and the shadows that are present in between the plants. The proposed system detects rice crops accurately, differentiates from other objects, and estimates the soil health based on plant counting data, which are approved by the aggrotech. The capability of the software is limited to a specified pattern; however, in the case of clusters, the count is semi-automated. Several attention-grabbing directions might be explored by future analysis in this domain. First, efforts could also be created to deploy our technique into an Associate in Nursing embedded system on a UAV for online yield estimation and exactitude agriculture applications. Second, the precise location of rice seedlings and, therefore, the practicability of rising investigation performance from the angle of object detection needs exploring; as a result, the precise location of crops may be a key step in exactitude agriculture. Third, since coaching knowledge square measure invariably the key to smart performance, particularly the range of such knowledge, it might be attention-grabbing to still enrich the RSC dataset as a precursor to more modeling work. Finally, the matter of object investigation in large-scale associations in nursing high-density environments continues to be an open issue, with lots of scope for exploring the practicability of rising investigation performance during this context.

## Figures and Tables

**Table 1:** Competitive analysis.

Reference	Crop Type	Technique	Limitation
[35]	Rice	Yolo v4	Low speed of target detection
[36]	Citrus trees	CNN	Less efficient for the densely populated region
[37]	Mango	Deep learning network	Less accurate
[34]	Rosette plants	L-system modelling	Confused with other objects required more data
[38]	Olive trees	U2 net- deep learning network	Complex model, error rate approx. 15%
Proposed Work	Rice	ARC-GIS, deep learning	Require good quality image for better object detection

**Table 2:** UAV and aerial inspection specifications.

Parameters	Specifications
UAV weight	1388 g
Max. flight time	10 min (approx.)
Battery	5870 mAh LiPo 4S
Flying altitude	35 m
Mission time	80 min (approx.)
Total flights	8

**Table 3:** UAV camera specifications.

Parameters	Specifications
Sensor	1″ CMOS effective pixels
Mechanical shutter speed	8–1/2000 s
Electronic shutter speed	8–1/8000 s
Photo	JPEG, DNG (raw)

**Table 4:** Imagery data set.

Agricultural Land	Region in Acres	Plantation Type	No. of Images
Region 1	35	Mechanical and manual	997
Region 2	5	Mechanical	203
Total	40	Mechanical and manual	1200

**Table 5:** Plant counting mechanically and manually.

Parameter	Value
Total Area	5 acres
Total Count	120,292 * plants
Mechanical Count	39,367 * plants
Manual Count	80,926 * plants
Total per Acre Count	27,488 plants
Mechanical per Acre Count	29,823 plants
Manual per Acre count	26,466 plants
Total Covered Area	4.38 acres
Mechanical Plantation	1.32 acres
Manual Plantation	3.06 acres

* Represents the approximation in Table 5.

**Table 6:** Plant counting mechanically.

Parameter	Value
Total area	35 acres
Total count	2,325,254 * plants
Per acre count	66,435 plants

* Represents the approximation in Table 6.

**Table 7:** Comparative analysis.

Reference	Technique	Efficiency
[34]	L-system modelling	95.00%
[35]	Yolo v4	98.84%
[36]	CNN	97.00%
[37]	Deep learning network	96.10%
[38]	U2 net- deep learning network	93.00%
Proposed Work	ArcGIS, Deep Learning	99.00%

**Table 8:** Proposed work outcome.

Technique	Study Area	Land Area	Experimental Count	Expected Count	Reason
UAV, Agisoft Metashape Professional software, ArcGIS, and Deep Learning	Rice	40 Acres	2,445,546	3,200,000 to 60,000,000	Soil is loam and baren

## Data Availability

Not applicable.
